# Preference and willingness to pay for nutritional counseling services in urban Hanoi

**DOI:** 10.12688/f1000research.10974.2

**Published:** 2017-11-15

**Authors:** Hai Viet Nguyen, Ngoc Bao Trinh, Huong Thi Le, Cuong Tat Nguyen, Hue Thi Mai, Tho Dinh Tran, Huong Thi Le, Quynh Ngoc Hoang Le, Bach Xuan Tran, Thuc Thi Minh Vu

**Affiliations:** 1 Institute for Preventive Medicine and Public Health, Hanoi Medical University, Hanoi, Vietnam; 2 Administration of HIV/AIDS, Vietnam Ministry of Health, Hanoi, Vietnam; 3 Institute for Global Health Innovations, Duy Tan University, Da Nang, Vietnam; 4 Department of Hepatobiliary Surgery, Viet-Duc Hospital, Hanoi, Vietnam; 5 Faculty of Pharmacy, Duy Tan University, Da Nang, Vietnam; 6 Bloomberg School of Public Health, Johns Hopkins University, Baltimore, MD, USA; 7 Department of Immunology and Allergy, National Otolaryngology Hospital, Hanoi, Vietnam

**Keywords:** Demand, willingness to pay, WTP, nutrition counseling services

## Abstract

**Background: **Despite substantial achievement in reducing malnutrition rates in Vietnam, there has been an increasing rate of overweight individuals in urban areas, which may result in a high burden of non-communicable diseases. Nutritional counseling clinics have been introduced in several settings; however, little is known about the preference for this service among urban clients. This study aimed to assess the preference and willingness to pay (WTP) for nutritional counseling services among urban clients.

**Methods: **We interviewed 429 clients who attended Hanoi Medical University Nutritional Counseling Clinic (Hanoi, Vietnam). WTP was determined using double-bounded dichotomous-choice questions and open-ended questions.

**Results: **In total, 78.6% respondents were willing to use nutritional counseling services. The mean amount of WTP for one-time service and one-year package was 96,100VND (~$4.3) and 946,400VND (~$41.9), respectively. Clients’ willingness to use the service was higher among females, those seeking counseling for elderly people and those who preferred face-to-face counseling services (p<0.05). WTP was higher among those who were over 35 years old, those seeking services for the elderly people, those having poor nutritional status, and those having under-6 year old children (p<0.05).

**Conclusions:** The preference and WTP for nutritional counseling services in urban Hanoi were relatively high. Scaling up this service is necessary to actively prevent and control the spread of non-communicable diseases.

## Introduction

In recent years, Vietnam has achieved a significant improvement in people’s health and nutritional status ^1^. This is indicated by an improvement in people’s knowledge, attitude and practice on nutrition, and a significant decrease in malnutrition rates among children. According to the National Institute of Nutrition, the rate of marasmus and stunting has been reduced from 19.9% (2013) to 14.1% (2015) and 32.6% (2013) to 24.6% (2015), respectively ^1^. However, in urban areas, significant increases in overweight and obesity rates may result in high burden of non-communicable diseases (NCDs) ^2^. A survey among 17,213 people in Vietnam showed that the rate of overweight and obesity was 16.3%. This high rate was fueled by unhealthy diet habits, alcohol abuse and sedentary lifestyles ^3^.

In developed countries, nutritional counselling has been recognized as an effective measure to improve awareness and encourage a healthy lifestyle, and has been shown to reduce the risk of obesity and NCDs ^4^. Nutritional counseling clinics can be organized in co-location with other general health care services or as stand-alone sites. However, in resource-scarce settings, this model has not yet to be implemented widely, due to the low responsiveness of health systems, as well as the poor practice of prevention against nutrition-related problems among the population ^5^. This condition can be seen in several countries around the world, such as Denmark or Western Australia ^5– 7^.

In Vietnam, nutritional counseling clinics have been recently introduced in metropolitan areas, including Hanoi and Ho Chi Minh City. However, little is known about the profile and preference of the clients that attend these clinics. To inform policy development and support the expansion of this service, the present study was conducted to assess the preference and willingness of clients to pay for nutritional counseling services in an urban site in Hanoi.

## Methods

### Study setting and sampling method

A cross-sectional study was conducted from March to April 2016 in an urban clinic in Hanoi Medical University, Hanoi, Vietnam. Eligibility criteria included 1) clients attending services in the Center of Preventive Medicine at Hanoi Medical University; and clients’ parents or guardians (for those who were under 18 years old); 2) aged 18 years and above; 3) agreed to participate in this study and gave written informed consent; 4) able to answer a questionnaire ( Supplementary File 1 and Supplementary File 2) for 15–20 minutes.

All eligible respondents from March to April 2016 were invited to participate in the study, resulting in a sample size of 429. 

### Measurements and instruments

**Socio-demographic variables** included age, gender, ethnicity, religion, educational attainment, marital status, current occupation, self-assessment of nutritional status and monthly household income (see Table 5 for detail).

**Preference** for nutritional counseling services included who would receive nutritional counseling, frequency of counseling services and communication methods for counseling.

**Willingness to pay** for nutritional counseling services were elicited using the bidding game technique, which consists of double-bounded dichotomous-choice questions combined with an open-ended question regarding two service packages: 1) fee-for-service; and 2) one-year nutritional management package.

**Figure 1.**
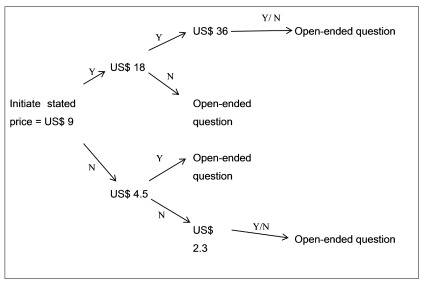
Bidding process to elicit the willingness to pay for one-time service.

**Figure 2.**
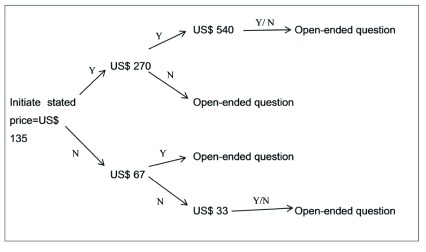
Bidding process to elicit the willingness to pay for one-year package.

We selected 200,000 VND (~ US$ 9; 2017 exchange rate) and 3,000,000 VND (~ US$ 135; 2017 exchange rate) to be the initial prices for fee-for-service and one-year nutritional management package, respectively, based on the actual price of nutritional counseling services in this clinic. Each patient was asked a series of questions about their WTP at specific prices (see Figure 1 and Figure 2 for the bidding process). Firstly, the clients were asked if they were willing to pay the initial prices. Depending on the choice of either Yes or No, interviewers presented two other bids: the higher bid for respondents answering “Yes”; and the lower bid for respondents saying “No”. The question was repeated until the last bid was equal to four times or one eighth of the initial prices. Finally, the respondents were asked an open-ended question “What is the maximum price you would be willing to pay for nutritional counseling services?”

### Statistical analysis

Data was analyzed using STATA software version 12.0 (Stata Corp. LP, College Station, TX, USA). A p-value <0.05 was considered statistically significance. A stepwise logistic model with the threshold of p-value < 0.2 was used to identify associated factors with the WTP. Interval regression was used to measure the amount of WTP and identify associated factors.

### Ethical approval

Proposal of this study was approved by the Ethical Committee of Hanoi Medical University. Subjects were introduced to the purpose of this study, and asked to give written informed consent if they agreed to participate in the study. Respondents could withdraw anytime they want. Their information was ensured to be confidential.

## Results

Demographic and socio-economic statuses of respondents are summarized in Table 1. Most of the clients were Kinh (97.7%), having above high school education (63.2%), single with no children (50.6–60.0%), and were in white collar employment (43.3%).

**Table 1.** Characteristics of respondents (n = 429).

Characteristics	Respondents								p-value
	Parents *		Male adult		Female adult		Total **		
	n	%	n	%	n	%	n	%	
**Ethnicity**									
Kinh	159	100.0	87	94.6	170	97.1	416	97.7	**0.02**
Other	0	0.0	5	5.4	5	2.9	10	2.4	
**Education**									
≤ High school	24	15	56	60.9	77	44	157	36.8	**<0.01**
> High school	136	85.0	36	39.1	98	56.0	270	63.2	
**Religion**									
No	156	98.7	86	93.5	172	98.3	414	97.4	**0.03**
Other	2	1.3	6	6.5	3	1.7	11	2.6	
**Marital status**									
Single/Divorced/Widow	15	9.4	76	82.6	125	71.4	216	50.6	**<0.01**
Live with spouse/partner	145	90.6	16	17.4	50	28.6	211	49.4	
**Employment**									
Freelance	26	16.7	8	8.7	12	6.9	46	10.9	**<0.01**
White collar	104	66.7	20	21.7	59	33.7	183	43.3	
Student	8	5.1	58	63.0	94	53.7	160	37.8	
Others	18	11.5	6	6.5	10	5.71	34	8.0	
**Have child under 6 years old**									
Single	20	12.5	80	87.0	156	89.1	256	60.0	**<0.01**
Yes	132	82.5	7	7.6	13	7.4	152	35.6	
No	8	5.0	5	5.4	6	3.4	19	4.5	

* Adults with children that were <18 years old. ** Some respondents refused to provide characteristic information, resulted in missing values.

Table 2 shows the willingness to use for nutritional counseling services of clients. Overall, 79.6% clients wanted to use counseling services. The major desire was that respondents’ children would receive nutritional counseling (74.8%) monthly or more frequently (39.8%) via meeting physicians face-to-face (64.9%).

**Table 2.** Preference for nutritional counseling service (n = 429).

Characteristics	Respondents								p-value
	Parents *		Male adult		Female adult		Total		
	n	%	n	%	n	%	n	%	
**Preference to use nutritional counseling services**	102	80.3	61	70.9	134	83.8	297	79.6	0.06
**Who should receive nutritional counseling services**									
Children - adolescents (<18 years old)	118	77.1	61	66.3	133	77.3	312	74.8	0.10
Adults (18–59 years old)	29	19.0	44	47.8	83	48.3	156	37.4	**<0.01**
Elderly (≥60 years old)	30	19.6	37	40.2	77	44.8	144	34.5	**<0.01**
No	31	20.3	12	13.0	15	8.7	58	13.9	**0.01**
**Frequency of receiving nutritional counseling**									
≤ Monthly	58	46.8	31	36.5	57	36.1	146	39.8	0.25
Every 3 months	31	25.0	27	31.8	59	37.3	117	31.9	
Every 6 months	21	16.9	20	23.5	30	19.0	71	19.4	
Every year	14	11.3	7	8.2	12	7.6	33	9.0	
**Communication methods**									
Face-to-face counseling	84	68.3	52	60.5	102	64.6	238	64.9	0.50
Telephone counseling	37	30.3	24	27.9	42	26.6	103	28.1	0.79
Mobile phone applications	13	10.6	23	26.7	28	17.7	64	17.4	**0.01**
Other	2	1.6	0	0.0	3	1.9	5	1.4	0.45
**Reason for not wanting to use nutritional counseling** **services**									
Comprehensive information on the Internet	9	10.0	9	17.0	17	21.5	35	15.8	0.12
Use this service elsewhere	0	0.0	4	7.8	3	3.9	7	3.2	**0.04**
Do not have money	2	2.2	5	9.4	8	10.3	15	6.8	0.08
Unnecessary	78	86.7	42	80.8	56	71.8	176	80.0	0.06
Other	11	11.0	5	8.9	12	14.1	28	11.6	0.62

* Adults with children that were <18 years old.

The WTP for one-time service is described in Table 3. Overall, a high amount of the respondents were willing to pay for nutritional counseling services (87.2%). The mean amount they were willing to pay was 96,100 VND per utilization (95% CI 81,000–111,000 VND), equivalent to US $4.3 in 2017, which varied across groups. There was a significant difference in the WTP of the three age groups (p<0.05).

**Table 3.** WTP for one-time service of nutritional counseling service.

Characteristics	One-time package			Amount of WTP		
	n	% ^a^	p-value	Mean	95% CI	
**Total**	259	87.2	-	96.1	81	111.2
**Gender**						
Male	65	87.8	0.85	100.6	65.5	135.7
Female	194	87.0		94.9	78.5	111.3
**Age**						
18–24 years	97	80.9	**0.03**	89.8	66.6	113.2
25–34 years	135	91.2		86.9	68.7	105.1
≥35 years	27	93.1		154.7	84.2	225.2
**Education**						
≤ High school	97	86.6	0.81	106.8	77.8	135.7
> High school	162	87.6		89.4	72.4	106.4
**Marital status**						
Single/Divorced/widow	136	85.5	0.36	100.2	78.7	121.8
Live with spouse/partner	123	89.1		92.8	71.3	114.2
**Employment ^b^**						
Freelance	32	86.5	0.23	112.4	57.2	167.5
White collar	105	88.2		84.1	64.1	104.1
Students	99	83.9		100.7	74.9	126.5
Other	21	100.0		128.3	61.9	194.8

^a^Percentage of 297 clients who responded to one-time service questions. ^b^Two clients’ employment statuses were missing.

Table 4 describes the WTP for the one-year nutrition management package. On average, respondents were willing to pay 946,400VND (95% CI 860,200 – 1,032,700 VND) (~$41.9 – 2017) for this package, which varied among groups (p<0.05).

**Table 4.** WTP for one-year package of nutritional counseling service.

Characteristics	One-year package			Amount of WTP		
	n	% ^a^	p-value	Mean	95% CI	
**Total**	173	46.5	-	946.4	860.2	1032.7
**Gender**						
Male	45	44.1	0.55	1027.7	823.5	1231.9
Female	128	47.6		918.7	827.0	1010.3
**Age**						
18–24 years	81	53.6	0.07	1059.5	912.0	1206.9
25–34 years	76	41.1		899.5	775.7	1023.4
≥35 years	16	44.4		756.7	609.5	904.0
**Education**						
≤ High school	76	53.5	**0.03**	1066.4	906.8	1226.0
> High school	97	42.2		878.0	778.1	977.9
**Marital status**						
Single/Divorced/widow	104	52.8	**0.01**	1002.6	888.4	1116.9
Live with spouse/ partner	69	39.7		893.7	764.3	1023.1
**Employment ^b^**						
Freelance	20	48.8	**0.01**	818.3	668.3	968.3
White collar	59	38.1		908.7	765.3	1052.1
Students	82	56.2		1067.3	919.4	1215.1
Other	11	50.0		777.3	585.0	969.6

^a^Percentage of 372 clients who responded to one-year package questions. ^b^One client’s employment status was missing.

Associated factors of the willingness to use and WTP for nutritional counseling services are shown in Table 5. The likelihood of using nutritional counseling services was higher among females, those seeking counseling for elderly people and those that preferred face-to-face counseling services. WTP for one-time service was 95,000 VND higher among clients aged over 35. Meanwhile, WTP for one-year nutritional management services was higher among those seeking services for the elderly people, those with a poor nutritional status and those that have under-6 year old children.

**Table 5.** Associated factors with preference and WTP for nutritional counseling services.

Characteristics	Willingness to use services		WTP for One-time package		WTP for One-year package	
	OR	95% CI	Coef.	95% CI	Coef.	95% CI
**Sociodemographic**						
Female (ref)						
Male	0.52**	0.28; 0.95				
**Age**						
18–24 years (ref)						
>35 years			95.78***	33.93; 157.61		
**Education**						
≤ High school (ref)						
> High school					-303.29**	-546.80; -59.79
**Household income** ^a^						
Poorest (ref)						
Rich			36.04	-13.58; 85.65		
Richest					197.18	-95.26; 489.63
**Have children under** **6 years old**						
Single (ref)						
Yes					266.30**	5.75; 526.84
No			74.24	-17.82; 166.30		
**Nutritional status ^b^**						
Very good (ref)						
Average			26.63	-8.23; 61.48		
Poor					635.65***	182.19; 1,089.11
**Target groups of** **counseling service**						
Children (ref)						
Elderly (≥60 years old)	1.82**	1.01; 3.27			261.72**	38.75; 484.69
**Communication** **methods**						
Face-to-face (ref)						
Telephone counseling	0.47**	0.26; 0.83				
**Constant**	4.12***	2.58; 6.60	38.10**	2.89; 73.32	823.58***	573.21; 1,073.96

*** p<0.01, ** p<0.05, * p<0.1 ^a^Household income: Poorest, ≤7,000,000VND/month (~$307.4); Poor, 7,000,000 – 10,000,000VND/month (~$307.4 – $439.2); Average, 10,000,000 – 15,000,000VND/month (~$439.2 – $658.8); Rich, 15,000,000 – 20,000,000VND/month (~$658.8 – $878.3); Richest, >20,000,000VND/month (~$878.3). ^b^Nutritional status (self-assessment of respondents), including: Very good; Good; Average; Poor; Very poor.

Raw data for Table 1–Table 5 Click here for additional data file. Copyright: © 2017 Nguyen HV et al. 2017 Data associated with the article are available under the terms of the Creative Commons Zero "No rights reserved" data waiver (CC0 1.0 Public domain dedication).

## Discussion

Nutrition has been a pressing topic of many researchers ^8^. There are several studies about nutritional counseling services for patients ^9– 11^ or concerning a particular nutritional component ^12, 13^, but studies about general and preventive nutritional counseling are still limited ^14^. Evidence provided by this study not only imparts information for future research, but also gives nutritional counseling providers a better perception to enhance their services.

In this urban setting, we found a high preference for nutritional counseling services for various target client groups, including elderly people and children. Clients also reported a high WTP for this service, which could be very helpful for expansion of the services. However, a combination of communication methods is needed; we found a higher preference for face-to-face counseling among respondents, knowing that many of them may also seek other health care services.

Overall, the preference for nutritional counseling in this study was quite high (79.6%). Most of the clients who did not have the need for this service were single with no children and self-evaluated their nutritional status as ‘average’. The mean amount of WTP for one-time and one-years services was $4.3 and $41.9, accounting for 0.20% and 1.98% GDP per capita in Vietnam in 2015 ($2,111, enumerated by World Bank) ^15^, which is an acceptable amount for clients to pay.

Associated factors of the preference and WTP for nutritional counseling services in our study were not in line with some predictions provided by a study in South Korea ^16^. Our study showed that older clients are more willing to pay for nutritional counseling than younger ones. Another noteworthy finding of this study is that clients with a higher educational level were not as willing to pay for the one-year management package as clients who only finished high school. This can be explained by the two occupations of respondents: those whose educational level were above high school were mainly white-collar workers, while almost everyone with lower educational levels were still high-school students or college students (83.8%). This may suggest that the recent nutritional education programs in Vietnam have caused a positive effect on students’ attitude toward nutritionally related programs ( http://dinhduonghocduong.net/) ^17^.

Those who have under-6 year old children and assess their children’s nutrition status poorly had a higher WTP for nutritional counseling services. These findings are well expected, thus enhance our study data’s validity. We suspected that clients’ income was associated with their WTP, as richer clients are more likely to pay a higher amount for nutritional counseling services. However, there was no significant relationship between clients’ household income and the WTP for nutrition counseling services.

To elicit a clients’ preference and WTP, we used the bidding game technique, as it was proved to be more reliable than open-ended questions or dichotomous-choice questions only ^18, 19^. However, one of the biggest drawbacks of this technique is that the risk of starting-point bias - the initial bid can have influence on clients’ WTP ^20^. The initial bids in this study were based on the actual prices for nutritional counseling services in this setting in order to minimize the occurrence of this bias. Additionally, our study may possibly be affected by other biases, such as observation bias, which occurs when the roles of respondents in their families can affect the amount of their WTP ^21^. For example, we assumed that those who were the bread-winners in their families tended to have higher WTP for health-related services. Another example is that if information about nutritional counseling may not be sufficiently provided, this may result in lower preference and WTP for nutritional counseling services. To mitigate this bias, we selected highly-experience interviewers and trained them carefully with a standardized protocol for data collection.

## Conclusions

The preference and willingness to pay for nutritional counseling services in urban Hanoi is relatively high. These findings may partly contribute to the implementation of maintaining nutritional counseling services Vietnam, thus actively preventing and controlling the spread of non-communicable diseases.

## Data availability

The data referenced by this article are under copyright with the following copyright statement: Copyright: © 2017 Nguyen HV et al.

Data associated with the article are available under the terms of the Creative Commons Zero "No rights reserved" data waiver (CC0 1.0 Public domain dedication). 

Dataset 1: Raw data for Table 1– Table 5. doi, 10.5256/f1000research.10974.d153260 ^22^

## Supplementary material

Supplementary File 1: Questionnaire in English.

Supplementary File 2: Questionnaire in Vietnamese.
