# Supplementary material for: Preference and willingness to pay for nutritional counseling services in urban Hanoi
Source: F1000Res. 2017 Nov 15;6:223. Originally published 2017 Mar 6. [Version 2] doi: 10.12688/f1000research.10974.2 (PMC5664972; doi:10.12688/f1000research.10974.2)
Supplement: Supplementary file 2 [file f1000research-6-13892-s0001.tgz › 02da5299-5805-483a-ab79-040ec9c809ed.docx]

| **A** | **GENERAL INFORMATION** | | | | | |
| --- | --- | --- | --- | --- | --- | --- |
|  | - **If clients are children under 18 y.o., enroll their guardian, and fill in both column A1.1 and A1.2** - **If clients are over 18 y.o., enroll the clients themselves, only fill in column A1.2** | | | |  | |
|  | 1. **RESPONDENT** | | 1. **CLIENT** | | | |
|  | **Date of birth**  **……………………...........** |  | **Date of birth**  **……………………...........** | | |  |
|  | **Sex**  Male  Female | 1  2 | **Sex**  Male  Female | | | 1  2 |
|  | **Ethnicity**  Kinh  Other (Specify):............... | 1  2 | **Ethnicity**  Kinh  Other (Specify):............... | | | 1  2 |
|  | **Height**  ............. cm |  | **Height**  ............. cm | | |  |
|  | **Weight**  .............kg |  | **Weight**  .............kg | | |  |
|  | **Religion**  No  Other | 1  2 | **Religion**  No  Other | | | 1  2 |
|  | **What is the highest level of education you have completed?**  Uneducated  Primary school  Secondary school  High school  College or technical school  University  Post graduate studies | 1  2  3  4  5  6  7 | **What is the highest level of education you have completed?**  Uneducated  Primary school  Secondary school  High school  College or technical school  University  Post graduate studies | | | 1  2  3  4  5  6  7 |
|  | **Marital status**  Single  Living with spouse  Living with partner  Divorced  Widowed | 1  2  3  4  5 | **Marital status**  Single  Living with spouse  Living with partner  Divorced  Widowed | | | 1  2  3  4  5 |
|  | **What is your current occupation? (with salary)**  Unemployed  Freelance  White collar  Farmer, worker  Student  Others (with salary) | 1  2  3  4  5  6 | **What is your current occupation? (with salary)**  Unemployed  Freelance  White collar  Farmer, worker  Student  Others (with salary)  Children | | | 1  2  3  4  5  6  7 |
|  | **What is your average monthly HOUSEHOLD income?**  ***(****Unit: million VND)* | | | | | …………… |
|  | **Currently, how many children do you have?** *(From the youngest to the oldest) (Only for married clients)*   \| **No** \| **Name** \| **Date of birth** \| **Sex** \| \| --- \| --- \| --- \| --- \| \| 1 \|  \|  \|  \| \| 2 \|  \|  \|  \| \| 3 \|  \|  \|  \| \| 4 \|  \|  \|  \| \| 5 \|  \|  \|  \| | | | | |  |
|  | **What was your age when you had your first child?**  *(Only for married clients)* | | | | | ............... |
| **G** | **NUTRITIONS – PREFERENCE FOR NUTRITIONAL CARE** | | |  | | |
|  | **Self-** **assessment of your/your child’s nutritional status**  Very good  Good  Average  Poor  Very poor | | | 1  2  3  4  5 | | |
|  | **Yesterday (for the last 24 hours), how many meals did you/ your child have (from last morning to this morning)?** ............... main meals .......... adjunct meals | | |  | | |
|  | **Frequency of comsuming these food yesterday (how many time did you eat these food yesterday?)**  Milk (not breastfeed)/ Dairy products  Starch /Rice  Meat/Fish/ Shrimp /Crab  Egg  Oil  Peanut/Sesame/Beans  Vitamin A rich vegetables (carrot, tomato, gooseberry leaf, broccoli, pumpkin)  Other vegetables  Fruits  Candy/Swet  Specify the kind of starch you ate:........................... | | | **Frequency**  ....................  ....................  ..................  ...................  ...................  ....................  ....................  ....................  ..................  ................... | | |
|  | **Who do you want to receive nutritional counseling in your family?** (multiple choices)  Children - adolescents (<18 years old)  Adults (18-59 years old)  Elderly (≥60 years old)  I don’t want this service | | | 1  2  3  4 🡪G23 | | |
|  | **How frequently do you want to receive nutritional counseling services?**  Daily  Monthly  Once per 3 months  Once per 6 months  Once per year | | | 1  2  3  4  5 | | |
|  | **By what method do you want to receive nutritional counseling?**  Face-to-face counseling  Telephone counseling  Mobile phone applications  Other:............................... | | | 1  2  3  4 | | |
|  | As you know, with the rise of the economy and society, unhealthy lifestyles took a detrimental effect on people’s health, such as overweight, obesity, malnutrition, cardiovascular diseases, gout…  To improve health status and alleviate the disease burden, we introduce the nutritional counseling services for each particular age groups (children, adults, elderly), provided by highly experienced physicians, answering quickly, completely and accurately all of your questions. | | |  | | |
|  | **Do you want to use this service?**  Yes  No | | | 1  2 🡪 G15 | | |
|  | **Do you want to use this service if it costs you some money?**  Yes  No | | | 1  2 🡪 G15 | | |
|  | **If the cost for nutritional counseling is 200,000VND per use, are you willing to use this service?**  Yes  No | | | 1  2 🡪G12 | | |
|  | **If the cost for nutritional counseling is 400,000VND per use, are you willing to use this service?**  Yes  No | | | 1  2 🡪 G14 | | |
|  | **If the cost for nutritional counseling is 800,000VND per use, are you willing to use this service?**  Yes  No | | | 1 🡪 G14  2 🡪 G14 | | |
|  | **If the cost for nutritional counseling is 100,000VND per use, are you willing to use this service?**  Yes  No | | | 1 🡪G14  2 | | |
|  | **If the cost for nutritional counseling is 50,000VND per use, are you willing to use this service?**  Yes  No | | | 1  2 | | |
|  | **What is the maximum amount that you can afford for one time service?**  *....................(thounsand VND)* | | |  | | |
|  | We also provide one-year nutritional management package, with cheaper price compares to one-time service. You can come to the Hanoi Medical University Nutrition Clinic to receive counseling anytime you want. | | |  | | |
|  | **Do you want to use the one-year nutritional management package?**  Yes  No | | | 1  2 🡪 G23 | | |
|  | **Who do you want to receive the one-year nutritional management package in your family?** (multiple choices)  Children - adolescents (<18 years old)  Adults (18-59 years old)  Elderly (≥60 years old) | | | 1  2  3 | | |
|  | **If the cost for one-year nutritional management package is 3,000,000VND per year, are you willing to use this service?**  Yes  No | | | 1  2 🡪G20 | | |
|  | **If the cost for one-year nutritional management package is 6,000,000VND per year, are you willing to use this service?**  Yes  No | | | 1  2 🡪 G22 | | |
|  | **If the cost for one-year nutritional management package is 12,000,000VND per year, are you willing to use this service?**  Yes  No | | | 1 🡪 G22  2 🡪 G22 | | |
|  | **If the cost for one-year nutritional management package is 1,500,000VND per year, are you willing to use this service?**  Yes  No | | | 1 🡪G22  2 | | |
|  | **If the cost for one-year nutritional management package is 750,000VND per year, are you willing to use this service?**  Yes  No | | | 1  2 | | |
|  | **What is the maximum amount that you can afford for one-year nutritional management package?**  *....................(thounsand VND)* | | |  | | |
|  | **Why don’t you want to use this service?**  Information available on the Internet  Used this service elsewhere  Economic-related issues  Not necessary  Other (specify)...................................... | | | 1  2  3  4  5 | | |

***THANK YOU FOR YOUR COOPERATION!***
