# Supplementary material for: Preference and willingness to pay for nutritional counseling services in urban Hanoi
Source: F1000Res. 2017 Nov 15;6:223. Originally published 2017 Mar 6. [Version 2] doi: 10.12688/f1000research.10974.2 (PMC5664972; doi:10.12688/f1000research.10974.2)
Supplement: Supplementary file 3 [file f1000research-6-13892-s0002.tgz › 0a197470-d58c-4486-98b9-a1154580e75f.docx]

| **A** | **THÔNG TIN CHUNG** | | | | | |
| --- | --- | --- | --- | --- | --- | --- |
|  | - **Nếu khách hàng là trẻ em dưới 18 tuổi, Hỏi người giám hộ đi cùng, ĐIỀN ĐỦ THÔNG TIN CỘT A1.1 và A1.2** - **Nếu khách hàng là trẻ em ≥ 18 tuổi hoặc người lớn, hỏi trực tiếp đối tượng, CHỈ CẦN ĐIỀN THÔNG TIN CỘT A1.2** | | | |  | |
|  | 1. **NGƯỜI TRẢ LỜI** | | 1. **NGƯỜI ĐƯỢC TIÊM CHỦNG** | | | |
|  | **Ngày/tháng/năm sinh:**  **.............** |  | **Ngày/tháng/năm sinh:**  **..................** | | |  |
|  | **Giới tính?**  Nam  Nữ | 1  2 | **Giới tính?**  Nam  Nữ | | | 1  2 |
|  | **Dân tộc**  Kinh  Khác (ghi rõ):............... | 1  2 | **Dân tộc**  Kinh  Khác (ghi rõ):............... | | | 1  2 |
|  | **Chiều cao**  ............. cm |  | **Chiều cao**  .............cm | | |  |
|  | **Cân nặng**  .............kg |  | **Cân nặng**  .................kg | | |  |
|  | **Tôn giáo?**  Không  Khác | 1  2 | **Tôn giáo?**  Không  Khác | | | 1  2 |
|  | **Anh/ chị đã hoàn thành hết cấp học nào?**  Không đi học  Cấp 1 - Tiểu học  Cấp 2 - Trung học cơ sở  Cấp 3 - Phổ thông trung học  Trung cấp, cao đẳng, dậy nghề  Đại học  Sau đại học | 1  2  3  4  5  6  7 | **Anh/ chị đã hoàn thành hết cấp học nào?**  Không đi học  Cấp 1 - Tiểu học  Cấp 2 - Trung học cơ sở  Cấp 3 - Phổ thông trung học  Trung cấp, cao đẳng, dậy nghề  Đại học  Sau đại học | | | 1  2  3  4  5  6  7 |
|  | **Tình trạng hôn nhân?**  Độc thân  Sống với vợ/ chồng  Sống với người yêu/bạn tình  Ly dị/ Ly thân  Góa | 1  2  3  4  5 | **Tình trạng hôn nhân?**  Độc thân  Sống với vợ/ chồng  Sống với người yêu/bạn tình  Ly dị/ Ly thân  Góa | | | 1  2  3  4  5 |
|  | **Hiện tại anh chị làm nghề gì? (có thu nhập)**  Thất nghiệp  Làm nghề tự do  Cán bộ, công chức, viên chức  Công nhân, Nông dân  Học sinh, sinh viên  Nghề khác (có thu nhập) ghi rõ | 1  2  3  4  5  6 | **Hiện tại anh chị làm nghề gì? (có thu nhập)**  Thất nghiệp  Làm nghề tự do  Cán bộ, công chức, viên chức  Công nhân, Nông dân  Học sinh, sinh viên  Nghề khác (có thu nhập) ghi rõ  Còn nhỏ | | | 1  2  3  4  5  6  7 |
|  | **Bình quân thu nhập của GIA ĐÌNH anh/chị là bao nhiêu tiền 1 tháng?**  ***(****Đơn vị: triệu đồng)* | | | | | …………… |
|  | **Hiện nay, gia đình anh/chị có bao nhiêu con?** *(Theo thứ tự từ nhỏ 🡪 lớn tuổi nhất) (Chỉ hỏi với những khách hàng đã lập gia đình)*   \| **STT** \| **Họ tên** \| **Ngày sinh** \| **Giới tính** \| \| --- \| --- \| --- \| --- \| \| 1 \|  \|  \|  \| \| 2 \|  \|  \|  \| \| 3 \|  \|  \|  \| \| 4 \|  \|  \|  \| \| 5 \|  \|  \|  \| | | | | |  |
|  | **Chị có con lần đầu tiên năm bao nhiêu tuổi?**  *(Chỉ hỏi với những khách hàng đã lập gia đình)* | | | | | ............... |
| **G** | **DINH DƯỠNG – NHU CẦU CHĂM SÓC DINH DƯỠNG** | | |  | | |
|  | **Anh/Chị đánh giá tình trạng dinh dưỡng của anh/chị/con mình như thế nào?**  Rất tốt  Tốt  Bình thường  Kém  Rất kém | | | 1  2  3  4  5 | | |
|  | **Ngày hôm qua (24 giờ qua) anh/chị/cháu ăn mấy bữa (từ khi ngủ dậy sáng qua tới khi ngủ dậy sáng nay):** ............... chính .......... phụ | | |  | | |
|  | **Tần suất tiêu thụ thực phầm ngày hôm qua (ăn mấy lần những TP sau)**  Các loại sữa (ngoài sữa mẹ)/ SP của sữa  Bột/gạo  Thịt/cá/tôm/cua  Trứng  Dầu/mỡ  Lạc/vừng/đậu đỗ  Rau/củ giàu Vit A (cà rốt, cà chua, rau ngót, súp lơ, bí đỏ)  Các loại rau củ khác  Quả chín  Bánh kẹo  Nếu ăn bột ăn liền ghi rõ loại bột:........................... | | | **Số lần ăn**  ....................  ....................  ..................  ...................  ...................  ....................  ....................  ....................  ..................  ...................  ................... | | |
|  | **Anh/chị muốn được khám và tư vấn dinh dưỡng cho đối tượng nào trong gia đình** (*chọn nhiều đáp án)*  Trẻ em – Trẻ VTN (<18 tuổi)  Người trưởng thành (18- 59 tuổi)  Người già (≥60 tuổi)  Không muốn SD DV này | | | 1  2  3  4 🡪G23 | | |
|  | **Anh/chị muốn được khám tư vấn dinh dưỡng tần suất như nảo?**  Hàng ngày  Hàng tháng  Hàng quý  6 tháng  Hàng năm | | | 1  2  3  4  5 | | |
|  | **Anh/chị muốn nhận dịch vụ khám tư vấn dinh dưỡng qua hình thức nào**  Gặp trực tiếp bác sĩ  Tư vấn qua gọi điện thoại  Qua ứng dụng điện thoại  Khác:............................... | | | 1  2  3  4 | | |
|  | Như anh chị đã biết, cùng với sự phát triển của kinh tế xã hôi, lối sống không lành mạnh đã gây ra rất nhiều vấn đề sức khỏe như thừa cân, béo phì, SDD, các bệnh như tim mạch, tiểu đường, gout...  Nhằm tăng cường sức khỏe nhân dân, giảm thiểu bệnh tật. Chúng tôi cung cấp dịch vụ khám và tư vấn dinh dưỡng cho các lứa tuổi (trẻ em, người trưởng thành, người già) bởi các **bác sỹ chuyên khoa nhiều kinh nghiệm**, giúp khách hàng được giải đáp nhanh chóng, đầy đủ, chính xác các vấn đề thắc mắc. | | |  | | |
|  | **Anh/chị có muốn sử dụng dịch vụ trên không?**  Có  Không | | | 1  2 🡪 G15 | | |
|  | **Nếu mỗi lần khám tư vấn dinh dưỡng mất phí anh/chị có sử dụng dịch vụ**  Có  Không | | | 1  2 🡪 G15 | | |
|  | **Chi phí dịch vụ là 200 nghìn/lần anh chị có sẵn sàng sử dụng không?**  Có  Không | | | 1  2 🡪G12 | | |
|  | **Chi phí dịch vụ là 400 nghìn/lần anh chị có sẵn sàng sử dụng không?**  Có  Không | | | 1  2 🡪 G14 | | |
|  | **Chi phí dịch vụ là 800 nghìn/lần anh chị có sẵn sàng sử dụng không?**  Có  Không | | | 1 🡪 G14  2 🡪 G14 | | |
|  | **Chi phí dịch vụ là 100 nghìn/lần anh chị có sẵn sàng sử dụng không?**  Có  Không | | | 1 🡪G14  2 | | |
|  | **Chi phí dịch vụ là 50 nghìn/lần anh chị có sẵn sàng sử dụng không?**  Có  Không | | | 1  2 | | |
|  | **Anh chị sẵn sàng trả nhiều nhất bao nhiêu cho 1 lần khám tư vấn dinh dưỡng?**  *....................(nghìn đồng)* | | |  | | |
|  | Ngoài ra, chúng tôi cũng cung cấp gói khám/tư vấn dinh dưỡng theo năm. Chi phí dịch vụ rẻ hơn. Bất cứ khi nào anh chị có nhu cầu có thể tới phòng khám dinh dưỡng tại trường Đại học Y Hà Nội để khám và tư vấn. | | |  | | |
|  | **Anh/chị có muốn mua gói khám/tư vấn dinh dưỡng cho cả năm không?**  Có  Không | | | 1  2 🡪 G23 | | |
|  | **Anh/chị muốn mua gói khám/tư vấn dinh dưỡng cả năm cho ai?** (nhiều đáp án)  Trẻ em (<18 tuổi)  Người trưởng thành (18 - 59 tuổi)  Người già (≥60 tuổi) | | | 1  2  3 | | |
|  | **Chi phí dịch vụ của gói này là 3 triệu/năm anh chị có sẵn sàng sử dụng không?**  Có  Không | | | 1  2 🡪G20 | | |
|  | **Chi phí dịch vụ là 6 triệu/năm anh chị có sẵn sàng sử dụng không?**  Có  Không | | | 1  2 🡪 G22 | | |
|  | **Chi phí dịch vụ là 12 triệu/năm anh chị có sẵn sàng sử dụng không?**  Có  Không | | | 1 🡪 G22  2 🡪 G22 | | |
|  | **Chi phí dịch vụ là 1.5 triệu/năm anh chị có sẵn sàng sử dụng không?**  Có  Không | | | 1 🡪G22  2 | | |
|  | **Chi phí dịch vụ là 750 nghìn/năm anh chị có sẵn sàng sử dụng không?**  Có  Không | | | 1  2 | | |
|  | **Anh chị sẵn sàng trả nhiều nhất bao nhiêu cho gói khám tư vấn dinh dưỡng này?**  *....................(nghìn đồng)* | | |  | | |
|  | **Tại sao anh chị lại không muốn sử dụng dịch vụ này**  Thông tin trên mạng rất nhiều  Đã sử dụng dịch vụ này tại nơi khác  Kinh tế không cho phép  Không cần thiết  Khác (ghi rõ)...................................... | | | 1  2  3  4  5 | | |

***XIN CHÂN THÀNH CẢM ƠN SỰ THAM GIA CỦA ANH/CHỊ!***
