# Supplementary material for: Preference and willingness to pay for nutritional counseling services in urban Hanoi
Source: F1000Res. 2017 Nov 15;6:223. Originally published 2017 Mar 6. [Version 2] doi: 10.12688/f1000research.10974.2 (PMC5664972; doi:10.12688/f1000research.10974.2)
Supplement: Peer review history

# Peer review: Referee response for version 2

**DOI:** 10.5256/f1000research.13892.r28479

**Published:** 2017-11-29

**Authors:** Wongsa Laohasiriwong

**Affiliations:** 1 Department of Public Health, Khon Kaen University , Khon Kaen, Thailand

I thank the authors for responding to my concerns in their comment. However as my original comment: 

 "I try to replicate the result by using STATA program,  but the result of the multiple logistic regressions is not the same those presenting in Table 5” 

 has not been addressed I have retained my original report status until such revisions are made.

I have read this submission. I believe that I have an appropriate level of expertise to confirm that it is of an acceptable scientific standard, however I have significant reservations, as outlined above.

# Peer review: Referee response for version 1

**DOI:** 10.5256/f1000research.11832.r24849

**Published:** 2017-10-30

**Authors:** Danielle Gallegos

**Affiliations:** 1 School of Exercise and Nutrition Sciences, Queensland University of Technology, Victoria Park Road,Kelvin Grove, QLD, Australia

Thank you for the opportunity to review this paper. Studies of this kind are vitally important as Viet Nam builds its infrastructure for the treatment and management of NCDs. The paper is interesting and technically sound. There is scope to expand the statistical analyses to control for a range of factors but only if these data were collected for example, BMI, diagnosis of an NCD, whether the child was underweight or overweight. 

 The only suggestion I have is to add to the discussion regarding the recent moves to train an expanded workforce in nutrition and dietietics. HMU is the first university to run the Bachelor of Nutrition degree and this expanded workforce will in the future build capacity for the delivery of nutrition counselling services in a range of settings. Knowing that clients are willing to pay for the service will contribute significantly to sustainability.  In addition, some discussion or speculation on whether this would negate the contribution of universal medical insurance would contribute to the discussion. 

 While I know that the review is meant to focus on the scientific content there are some content issues that need to be addressed 

 Abstract: I would be more general with the Dong described 96100 is too specific for the analysis performed and would be better cited as 96000 add in USD to the $ amount quoted.

 page 3 Western Australia is not a country and so this sentence needs to be adjusted.

I have read this submission. I believe that I have an appropriate level of expertise to confirm that it is of an acceptable scientific standard.

# Peer review: Referee response for version 1

**DOI:** 10.5256/f1000research.11832.r20978

**Published:** 2017-09-29

**Authors:** Wongsa Laohasiriwong

**Affiliations:** 1 Department of Public Health, Khon Kaen University , Khon Kaen, Thailand

The first, the topic is relevant and interesting. However, the authors need to clarify some points for future replication by other.

1.  Please confirm code of the dependent variable and independent variables in this study.I am not sure whether the dependent variable is Preference or WTP1 or WTP2.I try to replicate the result by using STATA program, but the result of the multiple logistic regressions is not the same those presenting in Table 5. 
  My analysis are: 
Table 1 
Table 2
Table 3 
2. Remarks on Table 5 mentions about the Vietnamese economic status using the monthly income quintile.  It would help us gaining better understanding if the authors discuss how the amount of 2,000,000 VND for single service and 3,000,000 VHD for yearly services related to their income status. Furthermore, if it is calculated as % of total expense, we will have better understanding on whether it is considered as high cost, or catastrophic cost or not.  However, for sure the expense for this counseling service is not too high. How could we guarantee that this could reflect the real perception of the value of counselling as priority and their willingness to pay when compare to other services such as treatment that have better short term output or outcomes.
  The methodology of biding to get the WTP amount is very interesting that the participants choose the amount themselves and use their mean as WTP. Please discuss concerning the amount that finally identify as WTP is appropriate in the real context of their living standard.

Participants of this research are clients who are seeking services at the Center of Preventive Medicine. Researcher should explain whether the sample could represent to population, since they might have some conditions that have higher demand for the services, then the results might be over estimate.

 Since this study is interesting:

1. Is the nutrition counseling fee  included in their service package (with all service) that they must pay. Or
2. The nutrition counseling is separate service that they could select or not select?

I have read this submission. I believe that I have an appropriate level of expertise to confirm that it is of an acceptable scientific standard, however I have significant reservations, as outlined above.

# Response

**Published:** 2017-10-01

**Authors:** Cuong Tat Nguyen

**Affiliations:** Institute for Global Health Innovations, Duy Tan University, Vietnam

Dear Prof. Wongsa Laohasiriwong,

Thank you very much for your review. I will provide explanations for your questions.

1. Both Preference, WTP1, and WTP2 are dependent variables, as they represented clients' willingness to use the nutritional counseling service, willingness to pay for one-time service and for a one-year package.

2. We selected 200,000 VND and 3,000,000 VND  to be the initial prices for the one-time service and one-year package, respectively, as they were the actual prices of nutritional counseling services in this clinic at that time. These prices were accounted for 0.43% and 6.4% of GDP per capita in Vietnam in 2015, yet still acceptable prices for Vietnamese clients to pay.

As for the comparison between this service and treatment, it is quite a hard question, since comparing the outcomes of preventive medicine and curative medicine is not an easy task. This could be a good idea for further studies about nutritional counseling service's outcome in the future.

3. The mean amounts of WTP for both one-time service and one year package are suitable for the living standard of Hanoi people, as this research was carried out in an urban area of Hanoi, with the majority of participants were students and white collar workers. These prices were also relevant to other medical services' prices such as out-patient examination (~150,000 VND) or chest X-ray (~80,000 VND).

4. We also have taken into account the risk of overestimating the preference of clients for nutritional counseling service, but this was not significant, as the participants were clients who sought other medical services in this clinic, e.g vaccination. The nutritional counseling service was a separate service and was not included in their medical package

Sincerely,

Cuong

# Peer review: Referee response for version 1

**DOI:** 10.5256/f1000research.11832.r20740

**Published:** 2017-03-09

**Authors:** Quan-Hoang Vuong

**Affiliations:** 1 FPT School of Business (FSB), FPT University, Hanoi, Vietnam

This research study has been well documented and technically sound, with regard to statistical consideration. The analysis employs standard techniques and sample size has been reasonable (N=429). The report of results should be welcome as the need for healthcare and related data has become increasingly imperative.

 The authors of the work have presented interesting findings, especially the significance of a small amount of spending, roughly $4 for one-time counseling service, and $42 for one-year package of services, both significant at conventional statistical levels. Apparently, there are rooms for the authors to report more insights since I can see the opportunity for controlling for some of the most influential socio-economic demographic factors, such as income, or physical state, such as BMI, where conditional probabilities computed from appropriate modeling efforts can potentially be insightful.

 I was also a bit surprised with the fact that the rate of approval for use of ICT apps and devices has been quite low, and this reality will be in and of itself an issue worth exploring as they are critically important in today's age of information, and help reduce counseling costs.

 All in all, this is a good and useful study, and I am happy to approve it for indexing by F1000Research.

I have read this submission. I believe that I have an appropriate level of expertise to confirm that it is of an acceptable scientific standard.
